# Sex differences in middle-aged and old Wistar rats in response to long-term sulforaphane treatment for prevention of neuroinflammation, cognitive decline and brain senescence

**DOI:** 10.1007/s10522-025-10231-0

**Published:** 2025-05-17

**Authors:** Roberto Santín-Márquez, Verónica Salas-Venegas, Jorge Antonio Garcia-Álvarez, Raúl Librado-Osorio, Armando Luna-López, Norma E. López-Diazguerrero, Beatriz Gómez-González, Mina Königsberg

**Affiliations:** 1 Departamento de Ciencias de la Salud, División de Ciencias Biológicas y de La Salud, Universidad Autónoma Metropolitana-Iztapalapa, A.P. 55-535, C.P 09340 Ciudad de Mexico, Mexico; 2 Aging and Metabolism Research Program, Oklahoma Medical Research Foundation, Oklahoma City, USA; 3 Facultad de Medicina, Unidad de Medicina Experimental “Dr. Ruy Pérez Tamayo”, Universidad Nacional Autónoma de México, Ciudad de Mexico, Mexico; 4 Facultad de Ciencias, Universidad Nacional Autónoma de México, Ciudad de Mexico, Mexico; 5 Instituto Nacional de Geriatría, SSA, Ciudad de Mexico, Mexico; 6 Departamento de Biología de la Reproducción, DCBS, Universidad Autónoma Metropolitana Iztapalapa, Ciudad de Mexico, Mexico

**Keywords:** Sex-dimorphism, Hormetin, Inflammation, Cellular senescence, Senomorphic, Cortex, Hippocampus

## Abstract

The nervous system (NS) experiences morphological and functional changes during the aging process, where low-grade chronic inflammation, oxidative stress and senescence are key regulators. Sulforaphane (SFN) is an isothiocyanate that activates redox response and inhibits the inflammatory process, which could modify the pro-inflammatory components of senescent cells secretory phenotype (SASP). Here we aimed to determine if SFN long-term treatment was able to prevent age-associated damage in the NS of adult and old females and males Wistar rats. We evaluated cytokines and chemokines profile, senescent cells markers, and memory parameters of adult (15 m.o.) and old (21 m.o.) rats after three months of SFN treatment. Young rats (4 m.o.) were used as age controls. Differences between sexes were observed in the inflammatory profile. Our results showed that SFN-treatment diminished proinflammatory molecules, senescence markers and senescent cells number in brain cortex and hippocampus from males and females’ adult rats, but no effects were observed in both sexes old groups compared with the same age control groups. SFN-dependent reduction in inflammatory and senescence parameters resulted in better scores in Barnes Maze Trial memory test when compared with same age non-treated group. Interestingly, adult females showed higher levels of proinflammatory cytokines and chemokines than adult males, which were prevented by SFN-treatment. No effects of SFN were observed in memory of old-treated groups.

**Supplementary Information:**

The online version contains supplementary material available at 10.1007/s10522-025-10231-0.

## Introduction

The increase in life expectancy has led to a rise in the incidence of age-related diseases. In particular, the nervous system is one of the most affected systems by oxidative stress and inflammation, which promote mental and motor changes during natural aging, affecting movement and cognitive performance (Aguilar-Hernández et al. 2023; Jurcau et al. 2024).

It is known that with aging there is a dysregulation of the immune system that generates a mild and persistent increase of proinflammatory molecules, producing a sterile and chronic inflammation, which has been termed “inflammaging” and stimulates the development of various chronic degenerative diseases (Franceschi, et al. 2000, 2018; Ferrucci & Fabbri 2018; Bleve et al. 2023). Another factor contributing to chronic low-grade inflammation during aging is the accumulation of senescent cells (Santoro et al. 2021; García-de la Rosa et al. 2024). Senescent cells are cells that irreversibly stop their proliferation in response to a stressor stimulus (Toussaint, et al. 2000; Rodier & Campisi 2011), and secrete a set of proinflammatory molecules known as SASP (Senescence-associated secretory phenotype (Rodier et al 2009; Maciel-Barón et al. 2018b; Gorgoulis et al. 2019). The SASP has both autocrine and paracrine effects, affecting the senescent cell itself as well as surrounding cells, and can induce different responses such as apoptosis of neighboring cells, reinforcement of the senescent state in the tissue, or proliferation of surrounding cells (McHugh & Gil 2018; Di Micco et al. 2021), thus altering the cellular microenvironment and therefore has been associated with the onset of various diseases. It has been reported that senescent cells in the brain contribute to neuroinflammation (Salas-Venegas et al. 2023; López-Teros et al. 2022, 2024) associated with cognitive deficit and neurodegenerative diseases (Nelke et al. 2022). Moreover, senescent cells have been reported in brains of patients with Alzheimer’s and Parkinson’s diseases (Bhat et al. 2012; Chinta et al. 2015). Several molecules capable of modifying the secretion of proinflammatory components of the SASP have been investigated. These molecules are referred to as senomorphics (Alimbetov, et al. 2016). Senomorphics are able to act at different levels of the secretory pathway, but molecules that additionally have other biological effects, such as modulation of the redox state or immunomodulation, like hormetins, had gained a clear advantage over other molecules that only have senomorphic effects. Hormetins are molecules (or specific conditions) that can induce health-beneficial effects in an hormetic manner (Rattan 2022); likewise, hormesis in the aging context is defined as the “phenomenon in which a repeated exposure to a stressor at low doses, that is toxic at high doses, induces a health-beneficial response” (Rattan 2008; Calabrese et al. 2019). One of these molecules with polypharmacological and hormetic activity is sulforaphane (SFN) (Guerrero-Beltran et al., 2012; Santín-Márquez et al. 2019; Calabrese and Kozumbo 2021; Fan et al. 2023). SFN is an isothiocyanate found in cruciferous vegetables and its administration has been linked to the activation of several molecular targets, most notably nuclear factor erythroid 2-related factor 2 (Nrf2) (Bai, et al. 2013; Briones-Herrera et al. 2018), but also inhibition of the activation of the NF-κB pathway, a master regulatory pathway of the inflammatory response, by inhibiting phosphorylation of inhibitor of κB (IκB) or directly preventing nuclear translocation of the NF-κB dimer (Pu, et al. 2018; Santín-Marquez et al., 2019). Our working group reported that SFN treatment reduced the release of proinflammatory SASP molecules in an in vitro model of senescent astrocytes derived from Wistar rats primary cortex, such as interleukin 1α (IL-1α), and increased anti-inflammatory molecules, such as interleukin 10 (IL-10), modifying the secretory profiles in senescent cells towards a preferentially anti-inflammatory profile (Maciel-Barón, et al. 2018a, b). SFN also showed anti-inflammatory and antioxidant properties, and it increased the secretion of glial trophic factors in hypothalamic astrocyte cultures, suggesting a glioprotective effect on aging brain (Santos et al. 2024). Others have reported that chronic administration of SFN in mice decreases cognitive impairment caused by hippocampal lesions similar to those found in Alzheimer’s development, and decreasing oxidative stress in the region in an Nrf2-dependent manner through the synthesis of antioxidant enzymes, such as heme oxygenase (HO-1) and NADPH quinone oxidoreductase 1 (Pu, et al. 2018).

In addition, in a previous study, we found that chronic (3-month) SFN treatment acted as a hormetin by improving redox status and preventing oxidative damage in middle-aged adult rats (15 months), but not in old animals (21 months). Although we found a beneficial effect in both females and males, adult females treated with SFN performed better in the evaluated parameters compared to males of the same age, and our multivariate analyses suggested that the two sexes responded differently to treatment (Santín-Márquez et al. 2022). One of the unresolved questions in the aging field is the heterogeneity in aging progression and phenotype, in particular sex-specific differences (Rattan 2024), therefore, in this study, we aimed to evaluate the effects of chronic SFN treatment on neuroinflammation and senescent cells accumulation in the brain, considering potential sex-specific differences, which in the future could help to generate more targeted and specific interventions.

## Material and methods

### Chemicals

All chemicals and reagents were purchased from Sigma Chemical Co. (St. Louis, MO). The reagents obtained from other sources are detailed throughout the text.

### Animals

The study was performed using young (4 months old), adult (12 months old), and old (18 months old) female and male albino Wistar rats (*Rattus novergicus*) housed in polycarbonate cages. All the female rats used were nulliparous. Four rats were housed per cage in a 12 h light–dark cycle, with ad libitum water and standard chow (Harlan Laboratories Inc. Indianapolis). Animals were provided by the vivarium of the Universidad Autónoma Metropolitana Unidad Iztapalapa (UAM-I). All procedures with animals were strictly carried out according to the National Institutes of Health Guide for the Care and Use of Laboratory Animals, the Principles of the Mexican Official Ethics Standard (NOM-062-ZOO-1999), and the Standard for the disposal of biological waste (NOM-087-ECOL-1995). The rats’ health status was evaluated to discard animals with tumors, skin, or ear infections. Euthanasia was performed by decapitation according to the NOM-062-ZOO-1999, section 9.5.3.3.

### Sulforaphane administration

Sulforaphane (SFN; LKT Laboratories, Cat. 4478-93-7) was dissolved in 1% DMSO and furtherly dissolved in saline solution (NaCl 0.9%). SFN was administered subcutaneously (0.5 mg/Kg, corresponding to 2.8 µM/Kg of body weight) five days a week for three months as previously reported (Miao et al. 2012; Santín-Márquez et al. 2022). Control groups were administered with the vehicle composed of DMSO 1% furtherly dissolved in saline solution (NaCl 0.9%) proportionally to body weight.

### Experimental groups

Individuals were sorted into 10 groups of 5 animals each, depending on sex, age, and treatment as follows: 4-month-old young females and males that were used as age controls and were not treated with SFN (n = 5). Adult females and males treated with SFN as mentioned above from 12 to 15 months of age, and their respective same-age control groups (n = 5). Old females and males treated with SFN from 18 to 21 months of age, and their respective same-age control groups (n = 3). Memory and learning tests were performed with a higher number of individuals, as follows: Females: Young: n = 10; Adult Ctl: n = 8; Adult SFN: n = 7; Old Ctl: n = 7; Old SFN: n = 6. Males: Young: n = 12; Adult Ctl: n = 10; Adult SFN: n = 7; Old Ctl: n = 6; Old SFN: n = 5.

### Biochemical assays

After euthanizing the brain was carefully extracted and washed with PBS 1X (Sigma-Aldrich, Missouri, USA; Cat. D5652). Brain cortex (Cx) and hippocampus (Hc) were dissected and stored in a − 70 °C ultra-freezer for further biochemical assays. Protein extraction was carried out as reported before (Santín-Márquez et al. 2022). Cx and Hc were mechanically homogenized with 800 µL and 400 µL, respectively, of lysis buffer (100 μL DTT 1 M, 100 μL Phenylmethylsulfonyl fluoride (PMSF) 0.1 M, 1 mini complete™ tablet (Roche, Switzerland) 10 mL T-PER™ Tissue Protein Extraction Reagent (Thermo Fisher Scientific, Massachusetts, USA; Cat. 78510) and furtherly centrifuged at 13,500 rpm at 4 °C for 15 min. Supernatants were separated and stored in aliquots at − 70 °C. Before each assay, protein concentration was determined by spectrophotometry at 595 nm, using a commercial Bradford reagent (Bio-Rad, Hercules, CA, USA).

### Cytokine determination

The determination was done using the ProcartaPlex® Multiplex Immunoassay kit (Thermo Fisher Scientific, Massachusetts, USA, Cat. EPX220-30122–901), which contains a panel of 14 rat cytokines (G-CSF, GM-CSF, IFNγ, IL-1α, IL-1β, IL-17 A, IL-2, IL-4, IL-5, IL-6, TNF-α, IL10, IL-12p70, IL-13) and 8 rat chemokines (Eotaxin, Gro-α, IP-10, MCP-1, MCP-3, MIP-1α, MIP-2, RANTES). The determinations were done according to the manufacturer protocol as follows, 50µL of standard and 30 µg of total protein samples were added to the 96 wells plate previously prepared with the detection beads and were incubated overnight at 4 °C. Then, 3 washes were performed before adding Detection Antibody Mixture, incubated for 30 min, and washed again 3 more times. After washes, 50 µL of Streptavidin-PE was added to the wells and incubated at room temperature for 5 min. After 3 more washes 120µL of Reading buffer was added to each well. The measurements were performed in a Luminex® Reader (Luminex, Canoga Park, CA) (Barajas-Gómez et al. 2017).

### Western blot

The Western blots were performed as reported by Hernández-Arciga et al., (2020). Briefly, 30 µg of protein samples were separated by SDS-PAGE using a 12% gel, after electrophoresis, proteins were transferred to PVDF membranes (Amersham Hybond™-P) in a Trans-blot® Turbo™ transfer system (Bio-rad, Hercules, CA). The unspecific union sites were blocked for 1 h at room temperature with non-fat dried milk 8% (w/v) in TBS-Tween. The membranes were incubated overnight at 4 °C with the primary antibody against γH2 AX (1:1000; Santa Cruz Biotechnology sc-517348), GLB1 (1:1500; Santa Cruz Biotechnology sc-65670), Lamin B1 (1:500; Abcam ab8982), p38 (1:2000; Abcam ab-31828) p21 (1:1000; Santa Cruz Biotechnology sc-6246), or β-actin (1:1000; Santa Cruz Biotechnology sc-47778) and washed with TBS-Tween. Afterward, the membranes were incubated in darkness for 90 min at room temperature with a horseradish peroxidase-conjugated secondary anti-mouse antibody (1:1000; Santa Cruz Biotechnology sc-35891) or anti-rabbit antibody (1:1000; Santa Cruz Biotechnology sc- 235) and washed 3 times with TBS-T. The protein bands were detected by chemoluminescence (InmobilonTM Western, Millipore, Billerica, USA). Densitometric analyses were performed using Kodak Molecular Imaging Software (v.4.5.1).

### Tissue preparation for histology

Rats were deep sedated with intraperitoneal 500 µL of sodium pentobarbital and perfused via the vascular system for 10 min with saline solution at a flux rate of 15 mL/min, followed by a solution of 4% paraformaldehyde and 1 M PBS at 4 °C for 10 min. The complete brain was extracted and stored for 4 h, at 4 °C in paraformaldehyde (4% in 1 M PBS). Then, PBS was retired and replaced by a sucrose solution (30%), agitated for 10 min at room temperature, and stored at 4 °C for 24 h. Afterward, the sucrose solution was retired and the tissues were embedded in 2-methyl butane and immediately put in dry ice until completely frozen and then embedded in Tissue Tek (Sakura Finetek, cat.4583). Fifteen µm thick coronal slices of the brain were obtained and mounted on the sides.

### Senescence-associated β-galactosidase activity determination

Brain slides were hydrated with 1 M PBS at room temperature in mild shaking for 10 min. Slices were permeabilized with PBS-Triton™ X-100 (Sigma-Aldrich, USA) 0.1% for 40 min in shaking and then washed for 3 times with 1 M PBS for 10 min. Slices were embedded in a work solution composed of 0.2 M citric acid, 100 mM ferrocyanide, 100 mM ferrocyanide, 5 M NaCl, MgCl_2_ 1 M, and X-gal 50 mg/mL (Promega, USA, cat. V3941), and incubated for 14 h at 37 ºC. After incubation, slices were washed 3 times with 1 M PBS and 25µL of Vectashield® mounting medium with DAPI (Vector Laboratories, USA, cat. H.1200) was added. Images were analyzed using the Zeiss ZEN 3.0 (blue edition) software. Cell numbers were normalized by 350μm^2^.

### Barnes maze trial.

The trial was performed using a 28 holes circular platform of 1.2 m in diameter located in a room with an intense white illumination which was used as an aversive stimulus to induce an escape behavior. The training was carried out for 4 days before the trial. On the first day, a habituation session was performed, in which the rats were allowed to explore the platform for 5 min. From the second to the fourth days two rounds of training were performed per day, with a lapse of 1 h between each training round. The escape tunnel was located only under one of the holes of the circular platform. During each training round, the rat was placed in the escape tunnel for 1 min. Then, it was placed in the center of the platform and was allowed to explore for 3 min or until it entered the escape tunnel. Finally, on the fifth day, the trial was performed. The time in which the individual found the escape tunnel was defined as “*latency*”, while the number of holes visited before the rat found the escape tunnel was defined as “*primary errors*.” Ten rounds of 3 trials each were performed per rat. During rounds 1–4, the escape tunnel was located in the same position as in the training rounds. At round 4 latency and primary errors were recorded and that determination was termed “*Spatial memory task*”. From rounds 5–7 the escape tunnel was relocated 120° from its original position; at round seven latency and primary errors were recorded and that point was named “*Learning task*”. From rounds 8–10 the escape tunnel was returned to its initial position; at round 10 latency and primary errors were recorded and that determination was named “*Working memory task*”.

### Statistical analysis

Univariate analyses were performed using Prism 9.5.1 (GraphPad Software, San Diego, CA, USA). All graphs were presented as mean ± standard deviation. D´Agostino-Pearson´s Omnibus test and Levene test were performed to determine the normality and homoscedasticity of the data. Since not all groups presented a normal distribution, the Kruskall-Wallis´ H test was applied followed by a Dunn post hoc test. *p* < 0.05 levels were taken as a significant difference between groups.

### Factor analysis

Owed to the high amount of variables, a factor analysis (FA) was performed in order to reduce the dimensionality of the variables and reclassify them into new factors which could accurately represent the variation in the model. An individual FA was performed for each brain region (Cx and Hc) and for the cognitive tests using “Maximum likelihood” as the factoring method and “Varimax” as the rotation method using the “factanal” function in R version 4.2.3 (R Core Team, 2023). Missing values were imputed for the analysis. Then, a Kaiser-Meyere-Olkin test (KMO) was performed to obtain the individual measure of sample adequacy (MSA) from each variable. Variables with an MSA < 0.50 were excluded from de FA. The Cx final overall MSA was equal to 0.77, the Hc final overall MSA was equal to 0.79, and the cognitive tests overall MSA was equal to 0.79. We continued performing a Bartlett’s sphericity test obtaining the following values: Cx: X^2^ = 876.16, *p* = 1.73e^−108^, df = 136; Hc: X^2^ = 1742.97, *p* = 5.10e^−108^, df = 276; cognitive tests: X^2^ = 135.73, *p* = 8.63e^−19^, df = 21. Afterward, the parallel analysis of the screen plot was performed to determine the number of factors required to describe the model. In all FA performed, 3 factors were sufficient, and the following values: Cx: X^2^ = 230.99, *p* = 8.89e^−15^, df = 88; Hc: X^2^ = 581.26, *p* = 2.96e^−37^, df = 207; cognitive tests: X^2^ = 10.21, *p* = 0.251, df = 8 were obtained. Loading plots and score plots were generated using JMP software version 16 (SAS Institute Inc, 2023).

### Discriminant analysis

All factors derived from the Cx, Hc, and cognitive tasks were used to perform a regularized discriminant analysis (RDA). In order to determine the best values for gamma and lambda a Cross-validation using the KlaR package in R version 4.2.3 (R Core Team, 2023) was performed. The final gamma value was equal to 0.00 and lambda was equal to 1.00. The discriminant analysis was performed using a stepwise variable selection in JMP software version 16 (SAS Institute Inc, 2023).

## Results

### Adult females exhibited higher levels of inflammatory molecules compared to adult males, but SFN treatment effectively reduced inflammation in both sexes

A 22 cytokines and chemokines panel was evaluated to determine the inflammatory state in the Cx and Hc of young, adult, and old females and male rats, treated with SFN or vehicle. The panel evaluated was classified into 3 groups as described as follows: pro-inflammatory cytokines (G-CSF, GM-CSF, IFNγ, IL-1α, IL-1β, IL-17 A, IL-2, IL-4, IL-5, IL-6, and TNF-α) (Supplementary Table 1 A & 1B); anti-inflammatory cytokines (IL10, IL-12p70, and IL-13) (Supplementary Table 2 A and B); and chemokines (Eotaxin, Gro-α, IP-10, MCP-1, MCP-3, MIP-1α, MIP-2 and, RANTES) (Supplementary Table 3 A and B). The concentration of IL-4, and MIP-2 obtained from both, Cx and Hc were below the detection ranks of the kit, so they were discarded from the analysis.

**Fig. 1:**
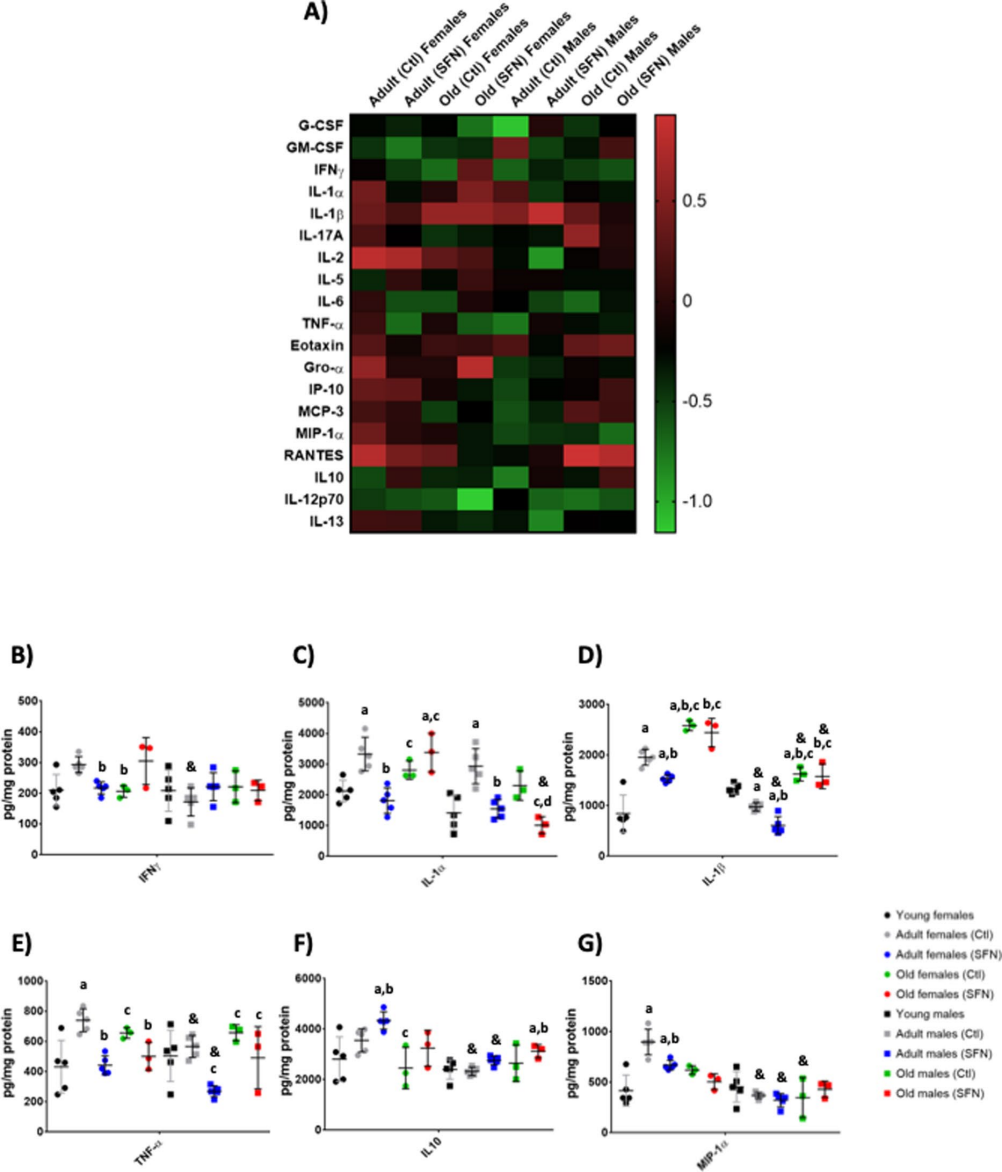
Cytokines and chemokines determination in the Cx of adults and old groups. The heatmaps show the complete cytokines and chemokines panels obtained for the cerebral cortex (Cx) of adult and old SFN-treated and untreated groups (**A**). Data were normalized against the same-sex young group concentrations (Supplementary Tables 1 A, 2 A, and 3 A). The concentration for the most representetive molecules in the Cx: IFNγ (**B**), IL-1α (**C**), IL-1β (**D**), TNF-α (**E**), IL-10 (**F**), and MIP-1(**G**). Each circle or square represents one animal. Young: n = 5; Adult Ctl: n = 5; Adult SFN: n = 5; Old Ctl: n = 3; Old SFN: n = 3. Kruskal–Wallis’s non-parametric test was performed, followed by Dunn´s multiple comparison test. Statistical significance as compared with same-sex young group (**a**) *p* < 0.05; against same-sex adult Ctl group (**b**) *p* < 0.05; against same-sex adult SFN-treated group (**c**)* p* < 0.05; and compared by sex (&) *p* < 0.05

**Fig. 2:**
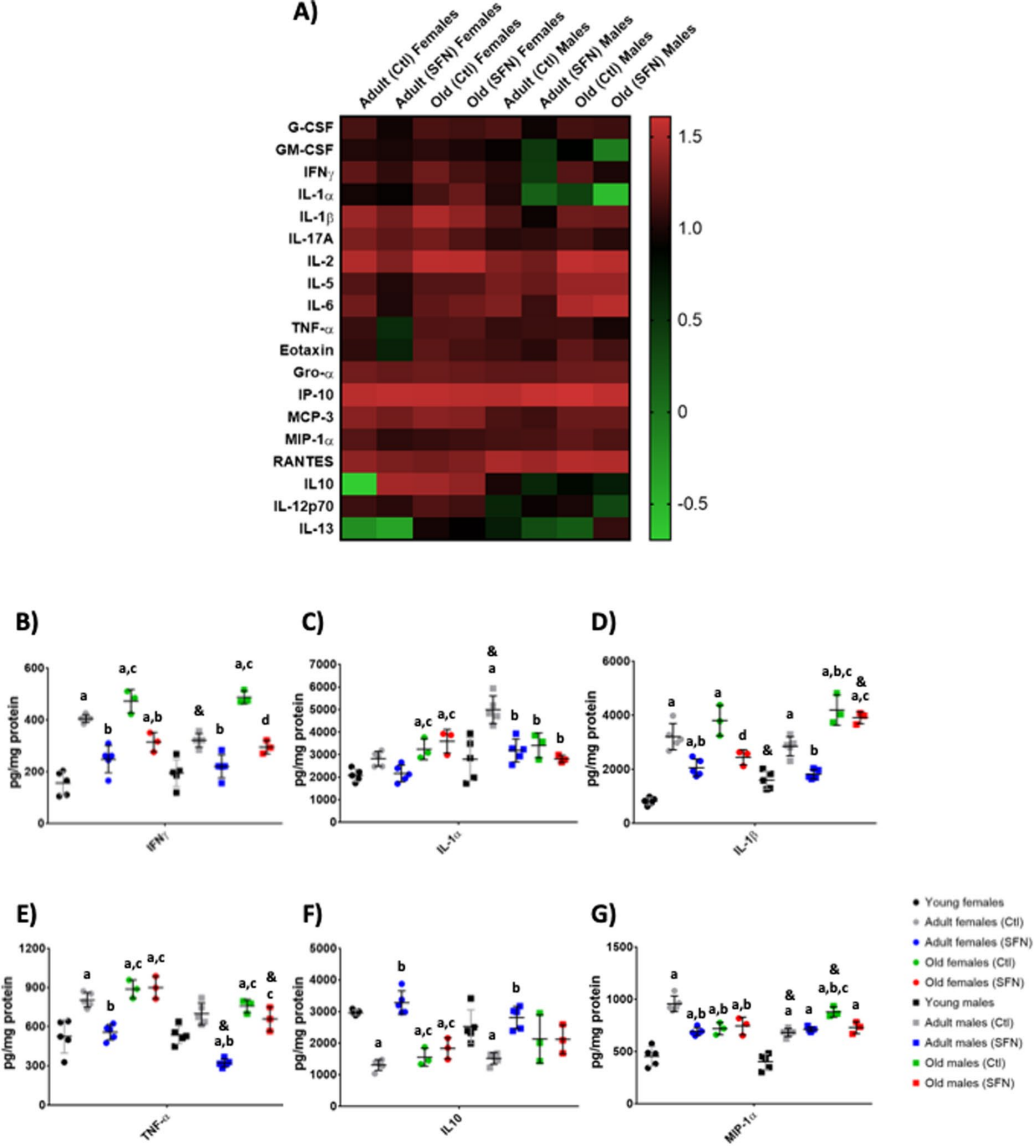
Cytokines and chemokines determination in the Hc of adults and old groups. The heatmaps show the complete cytokines and chemokines panels obtained for the Hipocampus (Hc) of adult and old SFN-treated and untreated groups (**A**). Data were normalized against the same-sex young group concentrations (Supplementary Tables 1B, 2B, and 3B). The concentration for the most representetive molecules in the Hc: IFNγ (**B**), IL-1α (**C**), IL-1β (**D**), TNF-α (**E**), IL-10 (**F**), and MIP-1(**G**). Each circle or square represents one animal. Young: n = 5; Adult Ctl: n = 5; Adult SFN: n = 5; Old Ctl: n = 3; Old SFN: n = 3. Kruskal–Wallis’s non-parametric test was performed, followed by Dunn´s multiple comparison test. Statistical significance as compared with same-sex young group (**a**) *p* < 0.05; against same-sex adult Ctl group (**b**) *p* < 0.05; against same-sex adult SFN-treated group (**c**)* p* < 0.05; and compared by sex (&) *p* < 0.05

The heatmaps show the complete cytokines and chemokines panels obtained for the Cx (Fig. 1A) and Hc (Fig. 2A) from adults and old groups; the data were normalized against the same-sex young group levels. Numerous changes in the inflammatory profile were observed in both sexes´ Cx and Hc during age and also after SFN treatment. Interestingly, higher levels of most cytokines and chemokines were found in the Hc than in Cx (Supplementary Tables 1–3). Most of the proinflammatory cytokines in the Cx of adult female rats (IFNγ, IL-1α, IL-1β, IL-17 A, IL-2, IL-6, and TNF-α) (Fig. 1B–E) were significantly increased with respect to young controls, but SFN treatment succeeded in maintaining them at young values in all cases except IL-2 (Supplementary Table 1 A). In contrast, no significant changes in these cytokines were determined in the Cx of male rats as they became adults. Treatment with SFN did not change their concentrations. Except for IL-1α which increased, and IL-1β which decreased, and, as in females, SFN prevented those effects (Fig. 1C and D).

Most cytokines in old animal Cx, regardless of sex, showed very similar concentrations to those in the young rats and did not change with the treatment (Supplementary Table 1 A). Notably, the most important difference was found between adult females and males, where females showed a significantly higher inflammatory state than males and young females.

In the Hc of female and male rats, a behavior similar to that of Cx was found in which cytokines increased upon reaching adulthood, and in the majority of the cytokines, SFN was able to prevent this increase (Supplementary Table 1B) (Fig. 2B–E).

Interestingly, IL-1α increased in adult males than in females, and the SFN treatment prevented this increase (Fig. 2C). In the case of the other proinflammatory cytokines, an increase in the concentration of most of them was also found in adult females, which was prevented by SFN. Less differences with respect to sex were found in the concentrations determined in Hc.

For the anti-inflammatory cytokines in the Cx, there was no significant decrease with age in either males or females, and SFN treatment only increased IL-10 in adult females and old males (Supplementary Table 2 A) (Fig. 1F). While in the Hc, IL-10 significantly decreased with age in females and SFN prevented this reduction in adult rats, but not in old rats (Supplementary Table 2B) (Fig. 2F). In males, the decreased only occurred in adults and was prevented by SFN. IL-12p70 augmented with age in females regardless of whether they were treated or not, and was unchanged in males; the same was observed for IL-13, which increased only in old females (Supplementary Table 2B).

When the chemokines concentrations were determined in females’ Cx (Supplementary Table 1 C), a similar pattern to in the pro-inflammatory cytokines was observed, where an increase during adulthood was noted and then a decrease at old age. A different behavior was observed in the females’ Hc chemokines panel (Supplementary Table 2 C), which augmented in the adult group, but no further reduction was determined during old age. Instead, the concentrations of the adult and the old females showed to be similar (Supplementary Table 2 C). SFN treatment did not show protective effects on most of the chemokines evaluated but did show protective effects on MIP-1α. This chemokine showed similar behavior in females’ Cx than eotaxin, Gro- α, and IP-10, which increased in the Cx of the adult group and then decreased in the old group. But in this case, SFN treatment did have a protective effect in adult females (Fig. 1G). The same occurred with MIP-1α in the Hc of adult females (Fig. 2G).

In summary, cytokines and chemokines panels in males and females were different. Pro-inflammatory cytokines in females’ Cx and Hc were higher than those concentrations obtained for males, mainly during adulthood. In the cases where cytokines increased in both sexes, SFN had a greater protective effect in males, but it is not excluded that it also managed to protect females, although to a lesser extent. It is interesting that in females, very varied effects were observed in the levels of these molecules with age, but not in males, which showed similar levels throughout life. This may be a relevant factor to explain sexual dimorphism during aging and the different responses to treatments and it will be discussed further.

### Senescent markers increase with age and sulforaphane treatment reduced their occurrence in middle-age

**Fig. 3:**
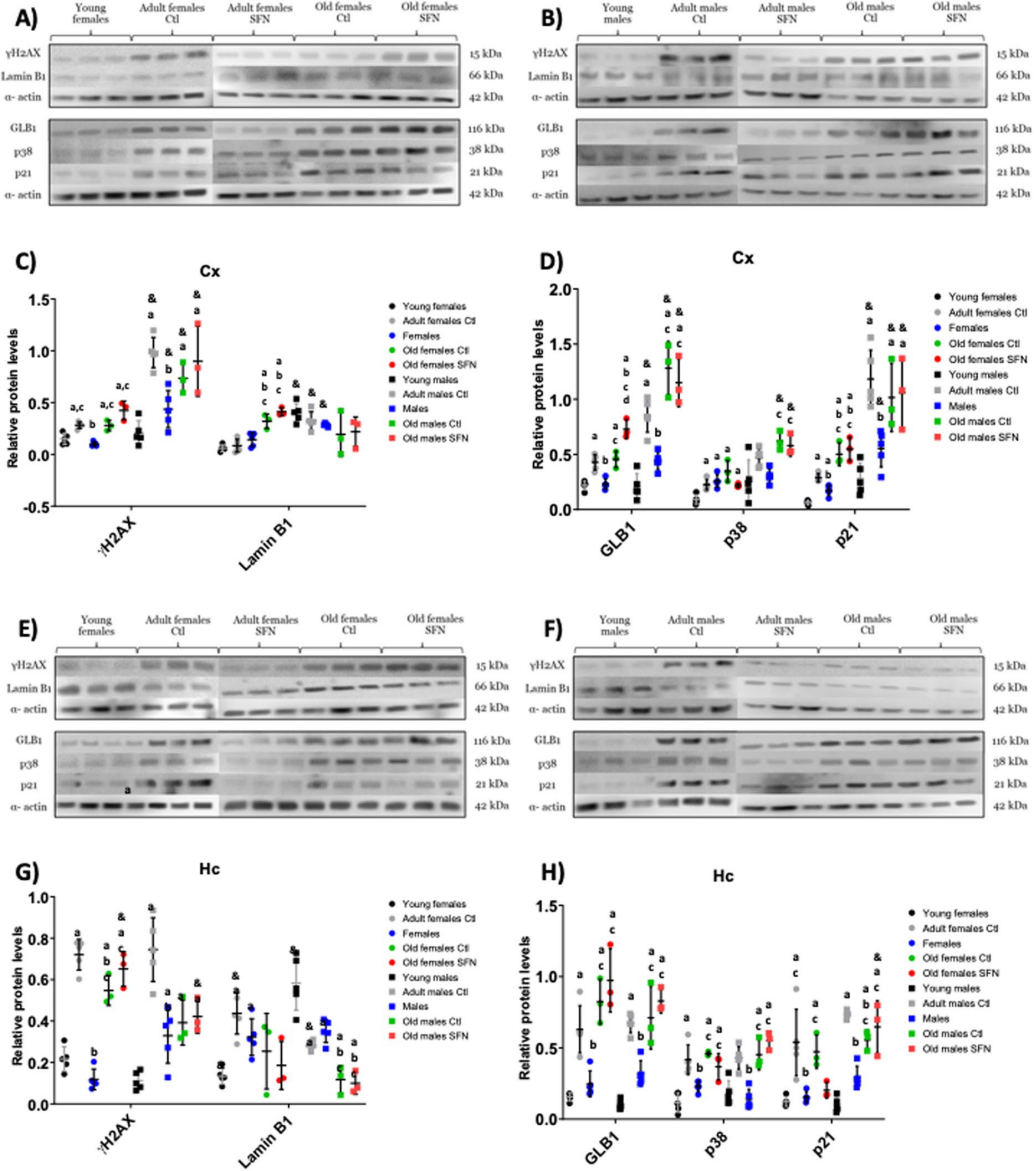
Senescent markers in the Cx and Hc of adults and old groups. Representative Western blots of senescent markers of adult and old SFN-treated and untreated groups: females (**A**) and males (**B**) in the Cx; and in the Hc (**E**) and (**F**). Densitometric analysis of each marker: γH2 AX and Lamin B1 in the Cx (**C**) and Hc (**G**); GLB1, p38, and p21 in the Cx (**D**) and Hc (**H**). Each circle or square represents one animal. Young: n = 5; Adult Ctl: n = 5; Adult SFN: n = 5; Old Ctl: n = 3; Old SFN: n = 3. Kruskal–Wallis’s non-parametric test was performed, followed by Dunn´s multiple comparison test. Statistical significance as compared with same-sex young group (**a**) *p* < 0.05; against same-sex adult Ctl group (**b**) *p* < 0.05; against same-sex adult SFN-treated group (**c**)* p* < 0.05; against same-sex old Ctl group (**d**)* p* < 0.05; and compared by sex (**&**) *p* < 0.05

To evaluate the abundance of senescent cells, some canonical markers were determined in the Cx and Hc, namely, γH2 AX, LaminB1, GLB1, p38, and p21. Although the amount of senescence markers increased with age in both sexes, it varied by brain region (Fig. 3). Higher levels of the senescence markers γH2 AX, GLB1, and p21 were found in males’ Cx in comparison to females’ Cx, in adult and old groups, regardless of SFN treatment (marked as & in Fig. 3). An age-dependent increase in γH2 AX, GLB1, and p21 was observed in males and females Cx. SFN treatment was capable of preventing the increase of these markers in the adult groups in comparison with their control groups (Fig. 3C and D). No changes were found in the old groups after the SFN treatment, except in the female old group, where GLB increased (Fig. 3D). For LaminB1 no differences were noted neither with age nor treatment in males and again the old female groups increased this marker regardless of the SFN treatment (Fig. 3C). P38 showed higher levels in adult and old groups, but no effects were found with SFN.

In regards to the Hc, the senescence markers found were similar in females and males of the same age and treatment (Fig. 3E–H), except for LaminB1 in males, contrary to females, decreased with age, and no differences were noted with SFN-treatment on adult or old groups (Fig. 3G). SFN treatment diminished the senescence markers in both sexes’ adult groups when compared with the same-age non-treated group, lowering them to similar levels to those observed in the young groups, suggesting that SFN might prevent senescence induction during adulthood in this region, but not at old age.

**Fig. 4:**
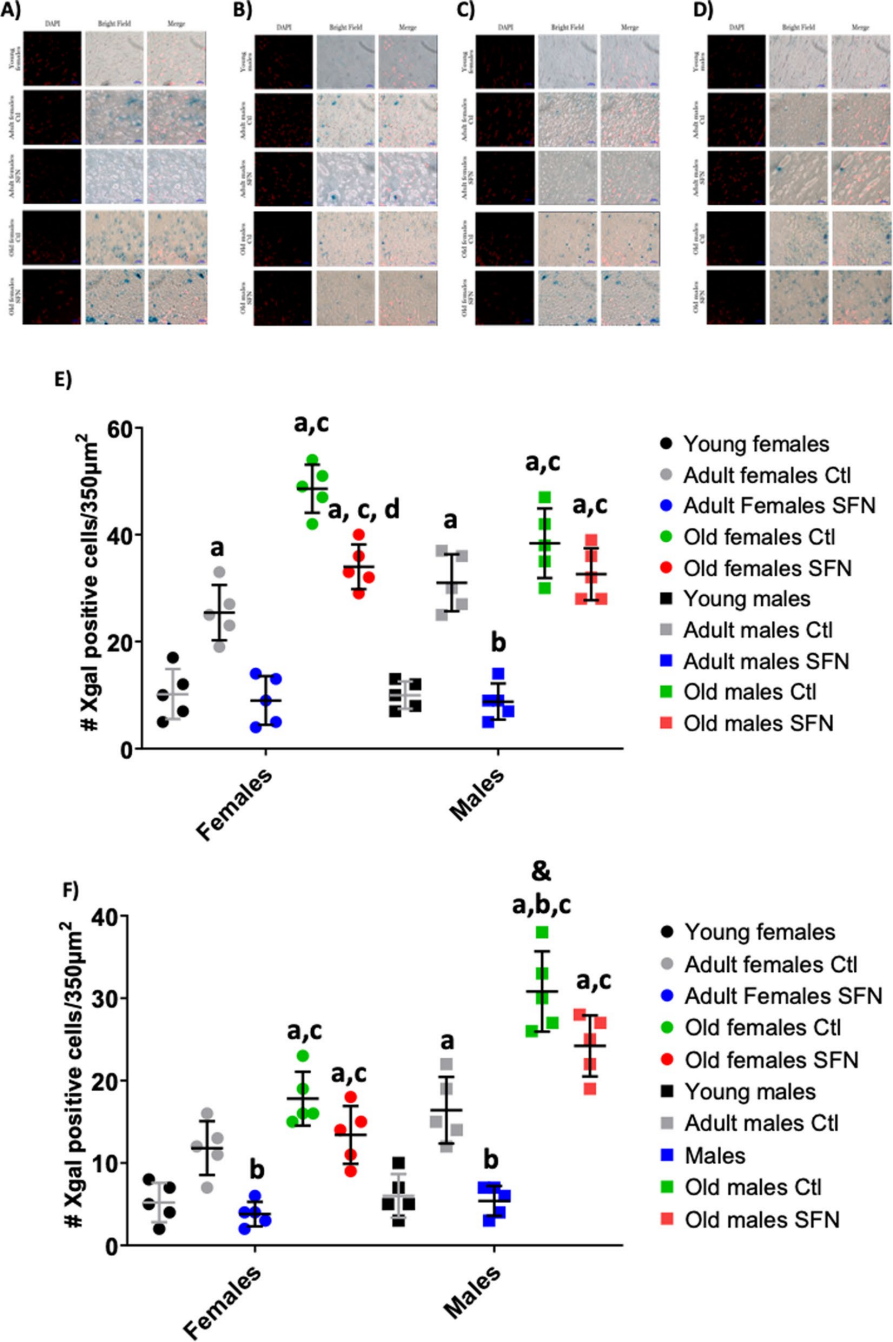
Positive cells to SA-β-Gal in the Cx and Hc of adults and old groups. Cells colored blue were counted as X-gal positive. Representative micrographs of adult and old SFN-treated and untreated groups: females (**A**) and males (**B**) in the Cx; and in the Hc (**C**) and (**D**). Number of X-gal positive cells/350 μm^2^ in the Cx (**E**) and Hc (**F**). Each circle or square represents one animal. Young: n = 5; Adult Ctl: n = 5; Adult SFN: n = 5; Old Ctl: n = 3; Old SFN: n = 3. Kruskal–Wallis’s non-parametric test was performed, followed by Dunn´s multiple comparison test. Statistical significance as compared with same-sex young group (**a**) *p* < 0.05; against same-sex adult Ctl group (**b**) *p* < 0.05; against same-sex adult SFN-treated group (**c**)* p* < 0.05; against same-sex old Ctl group (**d**)* p* < 0.05; and compared by sex (**&**) *p* < 0.05

An X-gal staining in brain slices was performed in order to determine the senescence-associated β-galactosidase activity and complement the senescence markers during senescence evaluation. An increase in X-gal positive cells was found with age in non-treated adults and old groups’ Cx and Hc regardless of sex (Fig. 4). SFN treatment prevented the increase in X-gal positive cells in both sexes of adult groups concurring with the findings in the senescent markers. In old groups, a lower number of X-gal positive cells was found in SFN-treated old females’ Cx in comparison to non-treated old females, no differences were observed in the rest of the old groups after SFN treatment (Fig. 4E and F).

### SFN prevented age-associated memory decline in a sex-dependent way

The Barnes’ maze trial was performed as described in the Methods section. We defined rounds 4, 8, and 10 as spatial memory tasks, learning tasks, and work memory tasks, respectively, since each block was designed to determine that particular cognitive skill. The latency and number of primary errors were assessed.

**Fig. 5:**
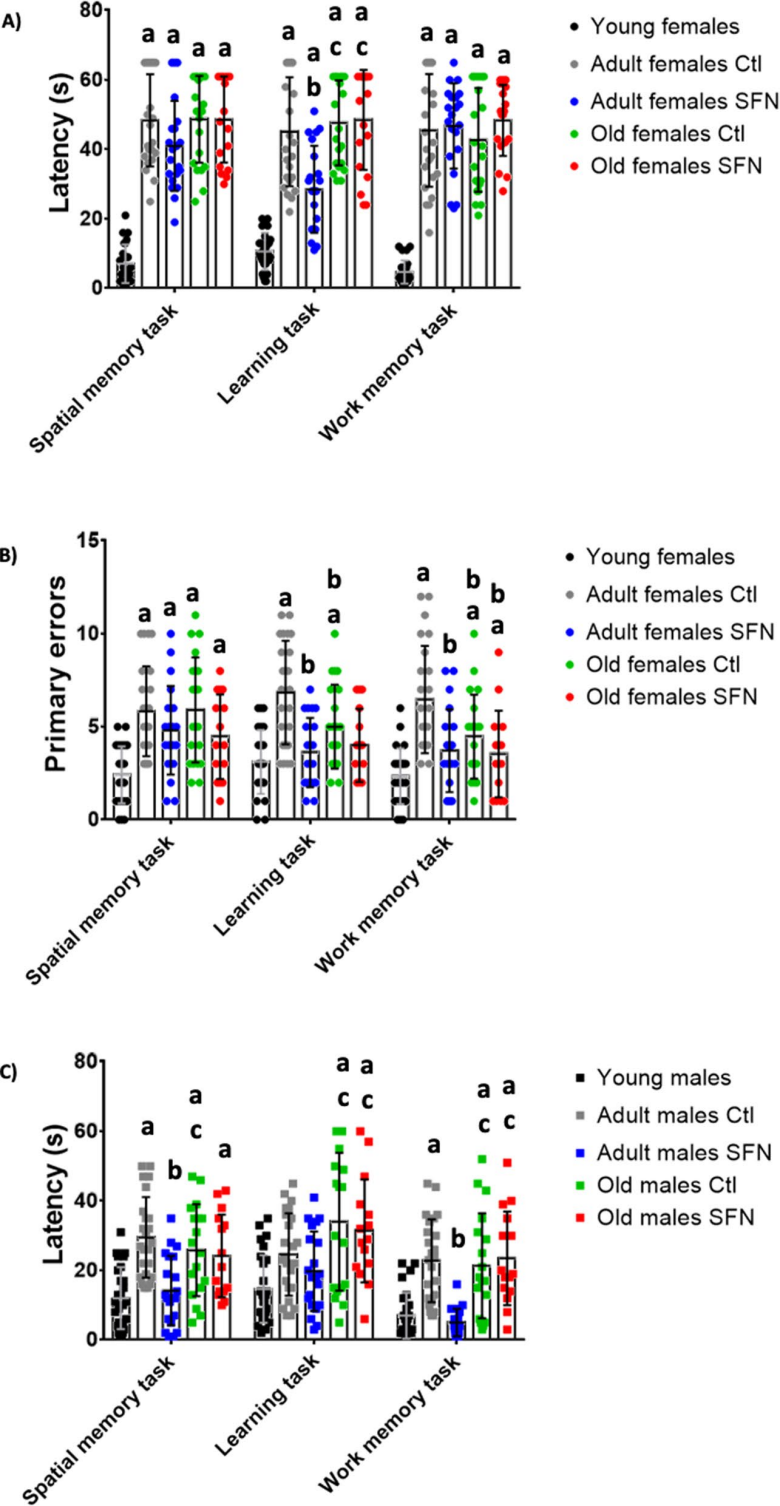

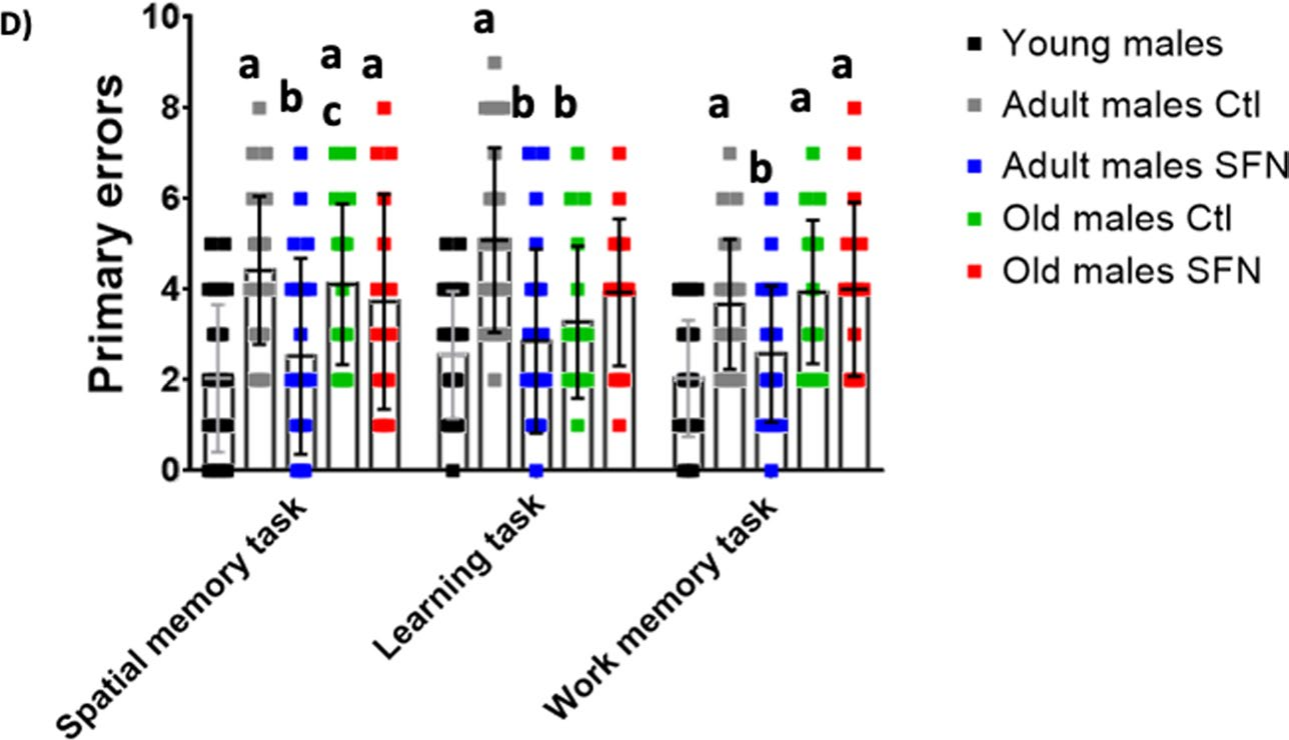
The Barnes’ maze cognitive test. Rounds 4, 8, and 10 were defined as spatial memory tasks, learning tasks, and work memory tasks, respectively. The latency for females (**A**) and males (**C**) was determined in seconds, along with the number of primary errors for females (**B**) and males (**D**). Each circle or square represents one animal. Young: n = 5; Adult Ctl: n = 5; Adult SFN: n = 5; Old Ctl: n = 3; Old SFN: n = 3. Kruskal–Wallis’s non-parametric test was performed, followed by Dunn´s multiple comparison test. Statistical significance as compared with same-sex young group (**a**) *p* < 0.05; against same-sex adult Ctl group (**b**) *p* < 0.05; against same-sex adult SFN-treated group (**c**)* p* < 0.05

In females, the latencies increased with age on average 4 times more in all three tasks (Fig. 5A), while in males the latencies increased, but not as much as in females (Fig. 5C). On average the latencies of adult and old male groups only doubled the latencies of the young males. No significant changes in spatial or working memory were determined after the SFN treatment, but lower latencies were found in the learning task in adult females compared to the non-treated adult rats. Conversely, adult-treated males showed significant differences in spatial and working memory tasks, in which latencies were lower in the treated adult males, but no differences were found in the learning memory task. Finally, old groups showed no differences after SFN treatment in any of the 3 cognitive tasks in both sexes.

Additionally to latency, primary errors were evaluated in the three cognitive tasks and were found to increase with age in both sexes (Fig. 5B and D). SFN treatment decreased primary errors in all three tasks in adult males, but only in learning and working memory in adult females. No differences were observed between treated and non-treated old groups regardless of sex.

### Factorial analysis

**Fig. 6:**
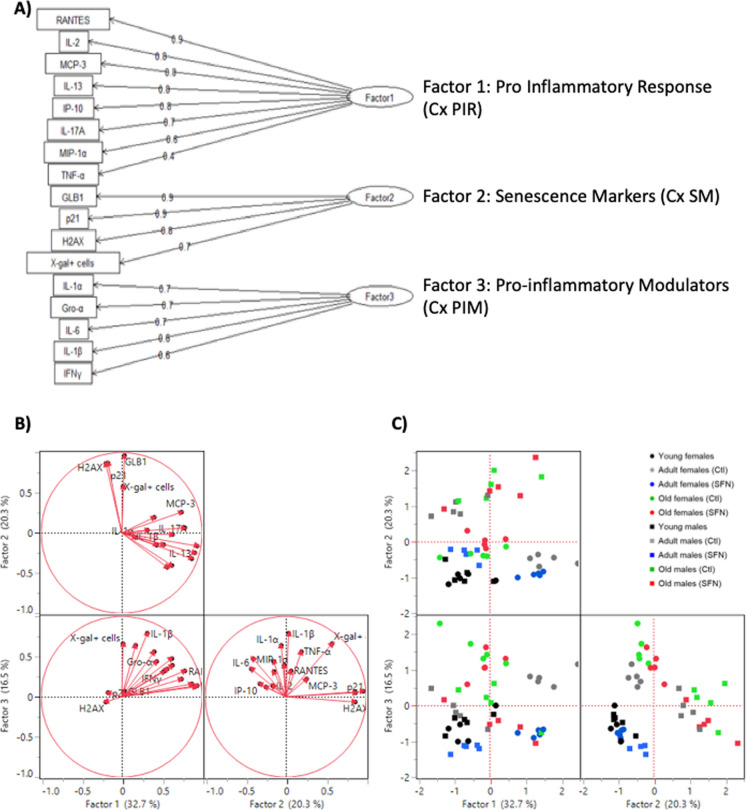

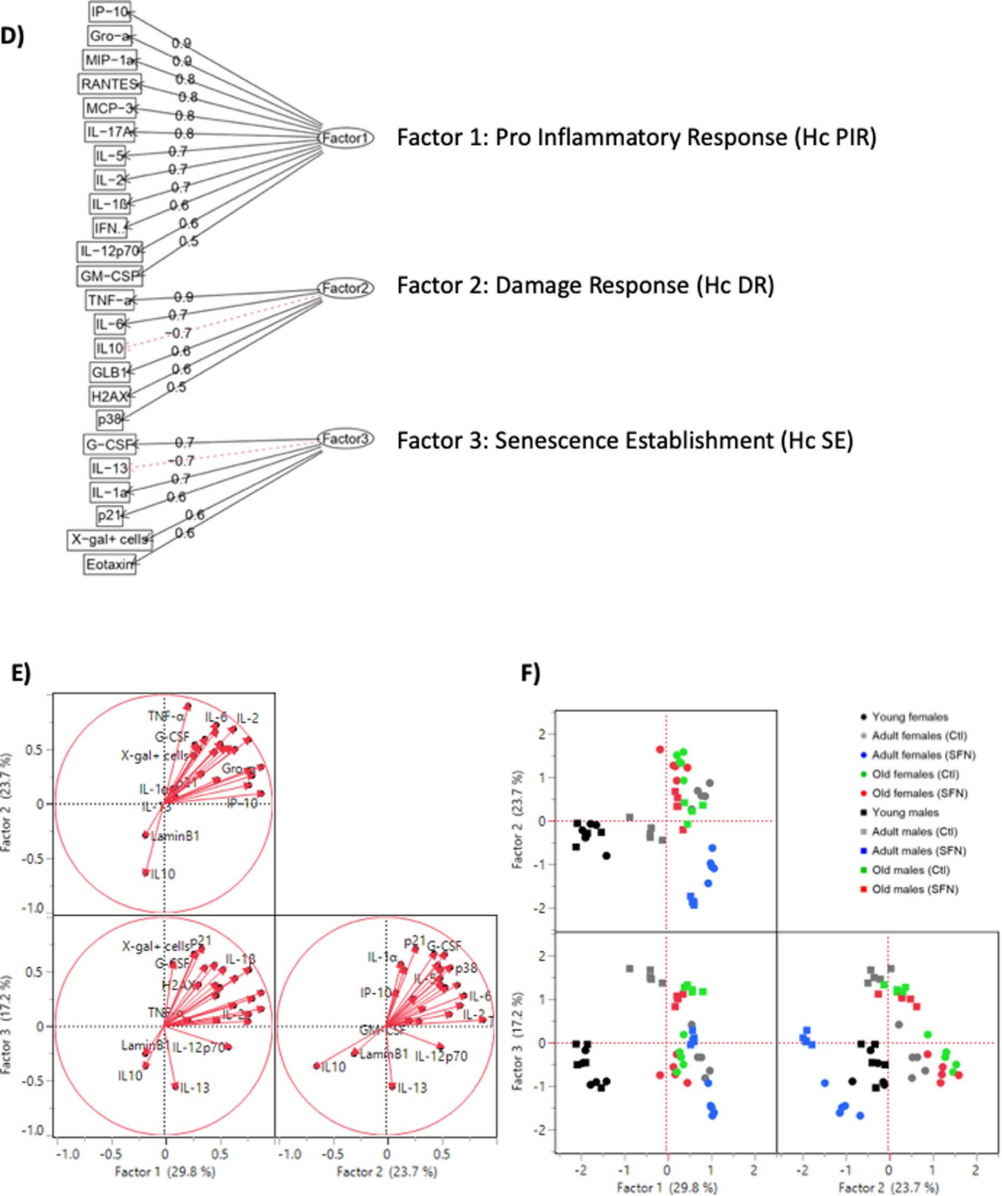
Factor analysis of Cx and Hc data. Tree diagram of the factor model used and factor´s given names for Cx (**A**) and Hc (**D**) data. All variables from both sexes were used to perform the factor analysis using the Kaiser-Meyere-Olkin (KMO) test; variables that had individual MSA (measure of sampling adequacy) < 0.50, were excluded from the analysis. Loading Plot for the Cx (**B**) and the Hc (**E**). Loads which represent each treatment (red dots), were plotted for rotated factors 1, 2, and 3. This helped to identify which variables have the largest effect on the factors. Loadings can range from − 1 to 1. Loadings close to − 1 or 1 indicate that the variable strongly influences the factor. Loadings close to 0 indicate that the variable has a weak influence on the factor. Score Plot for the Cx (**C**) and the Hc (**F**). The plots were performed using factors 1, 2, and 3, to detect clusters and trends of the data. Each point represents an individual. Circles represent females, while squares represent males

In order to reduce the number of variables and the dimensionality of our model without losing variation between them, we performed a factorial analysis (FA) as described in the materials and methods section. An independent FA was performed for the Cx and Hc, in which all cytokines, chemokines, and senescence markers were included. Also, we performed a FA for the cognitive parameters evaluated in Barnes’ maze trial. In a previous study using these same animals, our research team performed a novel object recognition test to evaluate discriminatory memory in adult and old rats under a long-term treatment with SFN (Santín-Marquez et al., 2022). SFN treatment had an effect on the discrimination index only in the adult groups, and no differences were found with respect to sex. Hence, to better understand this part of the phenomenon, we integrated these results to the FA. The factors obtained for each brain region were renamed according to the variables they contained to make the analysis clearer, as follows:

For the Cx, three factors that explained 69.5% of the total variation were obtained, (Factor 1 = 32.7%, Factor 2 = 20.3%, Factor 3 = 16.5%) (Fig. 6A and B). Factor 1 was named “*Pro-Inflammatory Response*” (**Cx PIR**) because it was positively related to RANTES, IL-2, MCP-3, IL-13, IP-10, IL-17 A, MIP-1α, and TNF-α concentrations. Factor 2 was named “*Senescence Markers*” (**Cx SM**), as it was related to GLB1, p21, and γH2 AX as well as to the number of X-gal positive cells. Finally, Factor 3 was named “*Pro-Inflammatory Modulators*” (**Cx PIM**) where IL-1α, Gro-α, IL-6, IL-1β, and IFN-γ were positively related. Figure 6C shows that in the Cx score plot, female and male young groups are negatively related to all three factors, being always located in the low left quadrant. The adult and old groups display differential spatial locations between females and males, since non-treated adult males were negatively related to Cx PIR, but positively related to Cx SM, while non-treated adult females were negatively related to Cx SM, but positively to Cx PIR. In both cases, SFN treatment reduced inflammation and senescence, but adult males had a better effect, as they were located closer to the young individuals. A higher data dispersion was observed for the old groups even though the majority were positively related to all three factors. Old males were strongly related to Cx PIR and Cx SM and old females were mainly related to Cx PIM, regardless SFN treatment.

In the Hc, three factors were obtained too, which explained 73.1% of the variation (Factor 1 = 29.8%, Factor 2 = 23.7%, Factor 3 = 17.2%) (Fig. 6D and E). As well as in the Cx, Factor 1 was termed “*Pro-Inflammatory Response*” (**Hc PIR**) because it contained similar cytokines and chemokines, and it was positively related to IP-10, Gro-α, MIP-1α, RANTES, MCP-3, IL-17 A, IL-5, IL-2, IL-1β, IFN-γ, IL-12p70, and GM-CSF. Factor 2 was called “*Damage Response*” (**Hc DR**) as it was positively related to TNF-α, IL-6, GLB1, γH2 AX, and p38 and negatively related to IL-10. Finally, Factor 3 was named “*Senescence Establishment*” (**Hc SE**) since it was positively related to G-CSF, IL-1α, p21, Eotaxin, and the number of X-gal positive cells and negatively related to IL-13.

The score plot for Hc presented in Fig. 6F shows a negative relation to all three factors in both sexes of young individuals. Non-treated adult males were strongly positively related to Hc DR and Hc SE while non-treated adult females were positive related to all three factors, showing a sex dependent difference, especially in Hc PIR. SFN-treated adult males and females responded differently to treatment because males were negatively related to Hc DR and not related to Hc SE, while females were negatively related to Hc DR and Hc SE and positively related to Hc PIR. Finally, old groups, despite SFN treatment, differed in Hc SE, to which males were strongly related while females were slightly negatively related. Similar locations were noted for factors Hc DR and Hc SE in both sexes’ old groups.

**Fig. 7:**
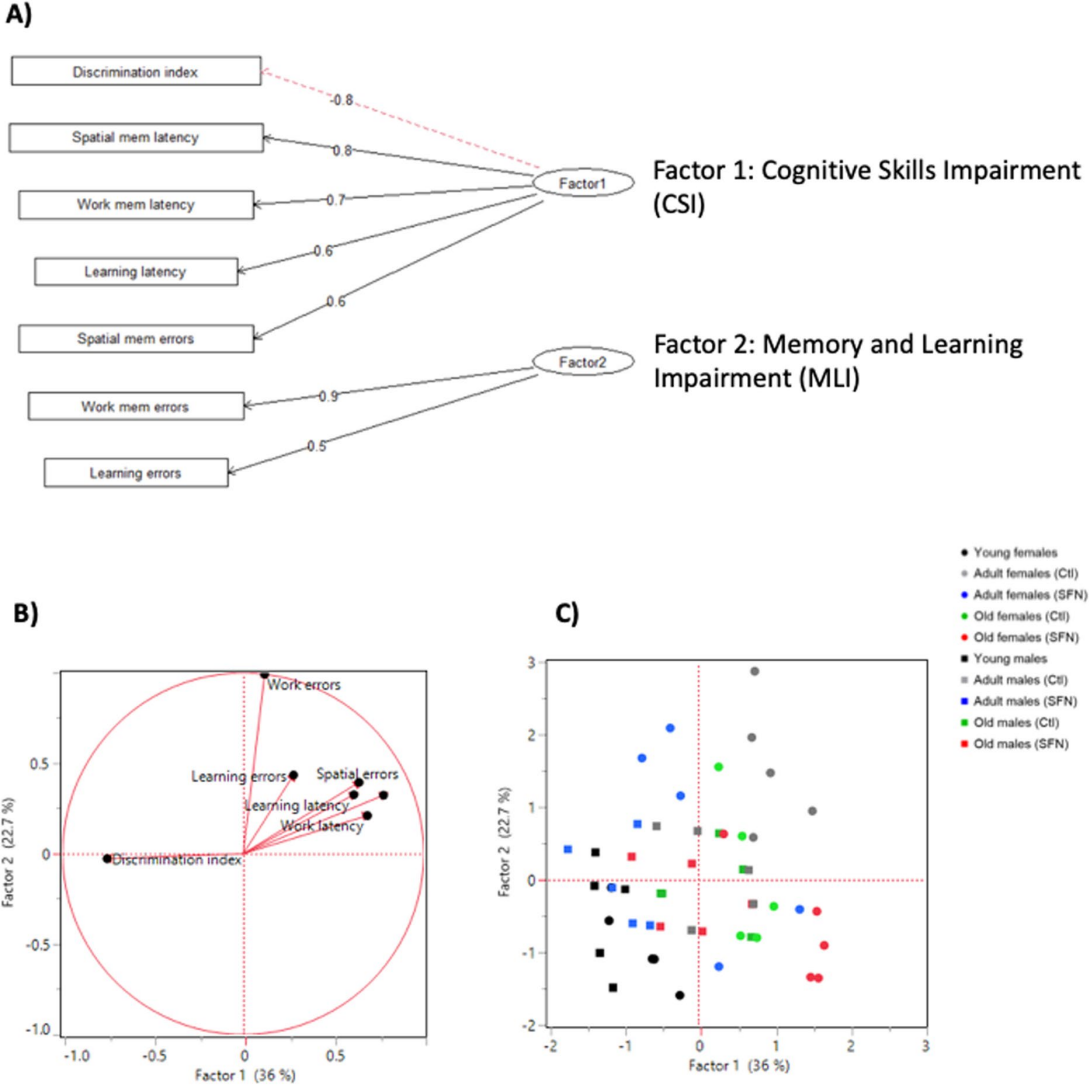
Factor analysis of the cognitive parameters. Tree diagram of the factor model used and factor´s given names (**A**). A FA was performed on the cognitive parameters, in which two factors were enough to explain 58.7% of the variation. Cognitive variables from both sexes were used to perform the factor analysis using the Kaiser-Meyere-Olkin (KMO) test; variables that had individual MSA (measure of sampling adequacy) < 0.50, were excluded from the analysis. Loading Plot for the cognitive parameters (**B**). Loads which represent each treatment (black dots), were plotted for rotated factors 1 and 2. Loadings close to − 1 or 1 indicate that the variable strongly influences the factor. Loadings close to 0 indicate that the variable has a weak influence on the factor. Score Plot for the cognitive parameters (**C**). The plots were performed using factors 1 and 2, to detect clusters and trends of the data. Each point represents an individual. Circles represent females, while squares represent males

Another FA was performed on the cognitive parameters, in which two factors were enough to explain 58.7% of the variation (Factor 1 = 36.0%, Factor 2 = 22.7%) (Fig. 7A and B): Factor 1 was strongly negatively related to the discrimination index and positively related to the spatial memory, working, and learning latencies and spatial memory number of primary errors, so it was appointed as “*Cognitive Skills Impairment*” (**CSI**). Factor 2 was termed “*Memory and Learning Impairment*” (**MLI**) since it was positively related to both working memory primary errors and learning primary errors.

In the cognitive parameters score plot shown in Fig. 7C, data dispersion was higher than in inflammatory and senescence FAs. As expected, both sexes’ young individuals were negatively related to CSI and MLI. Non-treated adult males were mainly located in the center of the plot, being weakly related to both factors, while non-treated females showed a strong relation to them, exposing a higher cognitive impairment. SFN-treated adult males were located closely to young males, thus showing a cognitive improvement. SNF-treated females improved their cognitive skills too, but to a lower extent in comparison to males, and with a higher dispersion. Finally, old groups were mainly located on the right side of the panel, being positively related to CSI and mildly related to MLI. SFN treatment was negatively related to both factors, presenting a slight improvement.

### Discriminant analysis

**Fig. 8:**
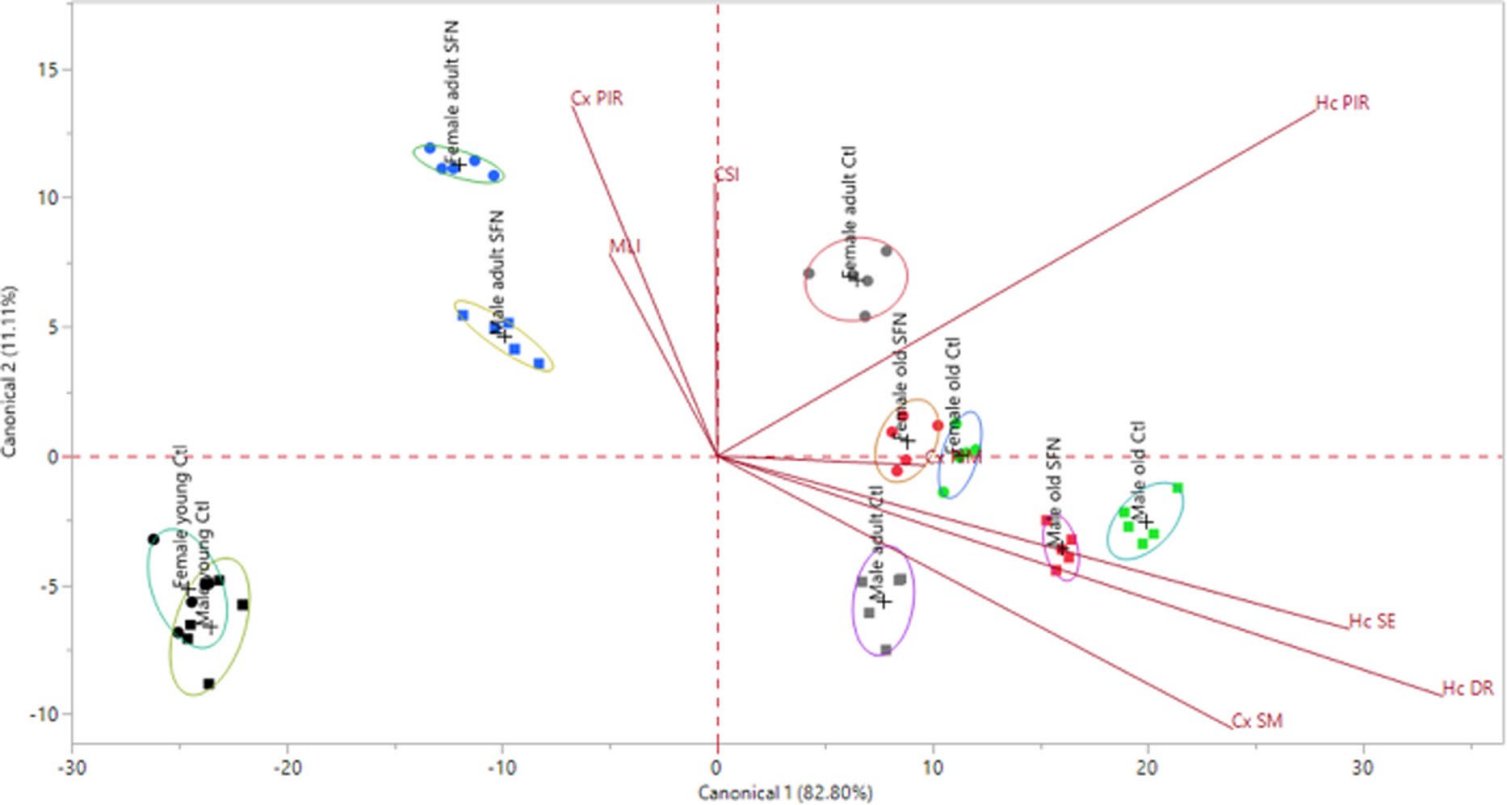
Discriminant analysis. The first and second canonical dimensions that best separate the groups are represented in the X and Y axis, respectively. The percentage of variation explained by each dimension is indicated. The length and direction of the biplot rays represent the level of association with each separated group. Each point represents an individual. Circles represent females, while squares represent males. Experimental groups are surrounded by 95% confidence level ellipses, dashed ellipses represent male groups and solid ellipses represent females. Group means are represented by a plus symbol (+). Statistically significant different groups’ ellipses do not intersect

All the factors generated in the FAs were used as covariates to perform a discriminant analysis, which was significant for our model (Wilk’s lambda = 3.74e-7, F_72,208_ = 30.05, p < 0.0001). Both canonical dimensions together explained 93.92% of the total variance of our model. The model is presented in Fig. 8, where each point represents an individual. Ellipses represent the 95% limit of confidence for the mean of each group. The image helps to have a visual representation of the phenomenon. When the ellipses overlap it means that the individuals in those groups behave in a statistically similar way, while if they are separated the individuals behave differently. The Biplot rays’ length and direction represent the association between groups and factors.

The young male and female groups were negatively associated with all factors; as they did not show cognitive impairment, inflammation, or cellular senescence, so they were placed in the lower left part of the panel. Adult and old groups displayed differences among sexes and treatments. Non-treated adults were located on opposite sides of the panel. Adult females without treatment were associated with PIR in both brain regions and cognitive impairment (MLI and CSI). In regards to non-treated adult males, they were positively related to Cx SM, Hc SE, and Hc DR, and negatively associated with cognitive impairment (MLI and CSI) and Cx PIR. Both sexes’ SFN-treated adults obtained a negative association with Cx SM, Hc SE, and Hc DR and were therefore located on the upper left side of the graph. SFN-treated adults showed an improvement in comparison to their respective non-treated group in the inflammatory and senescence-related factors. Adult males treated with SFN had a stronger change in the position of their group central point in comparison with females, which distance was lower.

For the old groups, the differences between SFN treatment and sexes were noted. A stronger association to Cx SM, Hc SE, Hc PIR, and Hc DR in old treated and non-treated males than females were observed, showing pro-inflammatory and senescence processes. Old female groups were located around the central part of the graph. A difference between SFN-treated old groups was noted too in female and male old groups. Even when the differences were discrete in comparison with the ones observed in adult groups, a slight improvement in the pro-inflammatory and senescence parameters was determined when compared with the non-treated old groups. The differences between sexes were conserved in adult and old groups, showing distinct associations to each factor.

## Discussion

It has been mentioned that the heterogeneity in aging progression and phenotype is a difficult and unsolved issue (Rattan 2024), and one of the problems is that sex inherent differences during brain natural aging were not considered as a determinant factor for the most part of the last century. Moreover, it is now known that neurodegenerative diseases are sexually dimorphic (Gemmati et al. 2019). For example, the female:male (F:M) prevalence of common neurodegenerative diseases is as follows: Alzheimer’s disease F:M 2:1; Parkinson’s disease F:M 1:1.5–2; amyotrophic lateral sclerosis (ALS) F:M 1:2; depression F:M 2:1; schizophrenia F:M 1:2. Interestingly, there is still a sex bias that favors studies with male patients or male animal models, as evidenced by the low percentage of females in clinical trials and female animals in preclinical studies in recent years (DuMont et al. 2023).

Since the decade of 1990’s an effort has been made, albeit insufficient, to promote studies evaluating the sex-dependent prevalence of several neurodegenerative diseases and natural brain aging (Cowell, et al. 1994; Salat, et al. 1997; Coffey, et al. 1998). Therefore, in this study we used female and male rats and found age- and sex-dependent differences in inflammatory mediators in adult and old groups. An increase in the levels of inflammatory molecules was evident with age in both sexes. The highest levels of inflammation-related molecules were reached during middle-age regardless the sex. However, interestingly, adult females got higher levels of cytokines and chemokines in the brain compared with same age males. The sexual dimorphism in inflammatory response has been has been defined as a multifactorial phenomenon (Klein & Flanagan 2016), where middle-age females have higher basal inflammatory levels than males in different brain regions (Cyr & de Rivero Vaccari 2023), resulting in differences in innate and adaptive immune responses along life (Klein, et al. 2010). It has been proposed that the sexual dimorphism in the basal neuro inflammation levels could be partially modulated by the differential proportion of specific microglia subpopulations which upregulate immune response which prevalently appears in females’ brain after middle age (Guillot-Sestier, et al. 2021). Nonetheless, some microglia subpopulations in males’ brain have been reported to increase the reactive oxygen species (ROS) production, as well as an increased activation and phagocytic activity (Doran, et al. 2019). A similar pattern has been described for astrocyte populations, in which activation of astrocytes is higher in the brain cortex and hippocampus of females than in the age-paired males (Murtaj, et al. 2019).

Middle age is a critical point to establish age-related inflammatory processes in both, females and males. In a detailed study, Mishra and colleagues (2020), showed the age-dependent change in a wide variety of genes related to the appearance of aging hallmarks, as metabolism regulators, grow factors dysregulation and senescence-related genes. Most of those genes changed during middle age in mice models and human samples, highlighting the importance and complexity of conserved biological processes which modulate the appearance of aging phenotype. However, inflammation is not the only process which is promoted during middle age. Processes as blood–brain barrier disruption and senescence appearance have been reported to increase during middle age too in murine models (Yamazaki, et al. 2016). SFN treatment prevented neuroinflammation in both sexes’ middle-age groups, showing better response in males than in females, probably owed to higher pro inflammatory state.

Although neuroinflammation is higher in females, we found higher levels of senescence markers and SAβ-gal positive cells in males when compared with female age-paired groups. This suggests sexual dimorphisms in the mechanisms underlying aging-related processes. Higher accumulation of senescent cells in males has been reported after middle age owed to factors as increase in oxidative stress and SASP soluble proteins. In an exhaustive study, Yousefzadeh and colleagues (2020) reported higher accumulation of senescence markers as p16^*Ink4a*^ and p21^*Cip1*^ in old males multiple tissues and organs compared with age-matched females. A possible explanation for higher senescence levels in males even when inflammation levels does not experiment a drastic increase during middle age as it occurs with females, could be related to oxidative stress more than inflammatory processes. It has been reported that females accumulate less ROS in brain compared with males, which produce higher ROS via mitochondrial electron transport chain and less efficient antioxidant mechanisms (Rubin, et al. 2020). In a previous study, we observed that middle age males had a more oxidative redox state, and the redox state parameters suggested that the first line antioxidant defenses were insufficient to contend with oxidative damage to macromolecules (Santín-Márquez, et al. 2022). Males exhibit an increase in senescence markers after pro inflammatory events, even when inflammatory markers were no different after insults (Schwab, et al. 2022), matching with the results obtained in this study.

Altogether, inflammatory processes and senescence establishment are increased with age in females and males, reaching its pike during middle age. Increase in both, inflammation and senescence have been reported to affect brain cognitive skills (Lin, et al. 2021; Hodges, et al. 2022). Moreover, as different patterns in levels of neuroinflammation and brain senescence were registered between sexes during middle age, differences in cognitive skills were noted as well. Middle-aged males’ spatial memory was better than age-matched females’. It has been reported that females experience cognitive decline earlier than males do, increasing the risk for some dementias and neurodegenerative pathologies (Levine, et al. 2021). Here, we observed and impairment in different types of memory in females during the middle age, which remains along elderly. It has been well characterized that males perform better in spatial memory tests than females (Safari, et al. 2021). And males usually maintain the integrity of spatial memory during aging, probably owed to a better integrity in hippocampal neuron connectivity and plasticity (Jacobson, et al. 2008). Even though males and females had a clear difference in spatial memory, no differences in short term memory had been registered. SFN treatment was able to prevent cognitive decline in middle age in males, but partially done it with females.

As we described above, differences by sex, age and treatment were registered in inflammation, senescence and memory parameters. In order to reduce the dimensionality of the study and integrate variables into a less complex system, we performed a FA for each brain region. We observed different distribution in the plane for females and males, being located in separated individual groups. It showed that, effectively, males were positively related to damage response and senescence factors, while females are positively related to pro inflammatory responses. This suggests different mechanisms underlying neurodegenerative processes related to aging progress in a sex-dependent fashion. These sex-dependent mechanisms are not only influenced by sexual hormones presence, but by a wide variety of molecules secreted by immunomodulatory cells. Particularly speaking of the brain, glial cells have a key role in the progression of many pathological and physiological events within the brain. Astrocytes and microglia regulate of brain age-related damage. It has been described that females have more microglial cells than males do, as well as higher microglial secretion of pro inflammatory cytokines (Murtaj, et al. 2019; Lynch 2022). This correlates with higher levels of microglial activation and transcription of M1 state markers in aged female mice than in aged males (Han, et al. 2021), and undergo faster to activated state than same age males (Frigerio, et al. 2019). In regards to sex differences in astrocytes, in was observed that males’ astrocytes have increased phagocytic activity after inflammatory processes to recover homeostasis, while females decreased the phagocytic activity and increased inflammatory phenotypes (Crespo-Castillo et al., 2020).

It is very interesting that the effect of a hormetin that also acts as a senomorph is different in males and females. As mentioned before, in a previous work with the same groups of animals and the same SFN treatment, we found that females showed better protection in terms of redox state and oxidative damage, and that the mechanism by which they were protected seemed to be different from that of males (Santín-Márquez, et al. 2021,  2022), but here we found that the levels of proinflammatory cytokines in the brain of adult female rats increased with age compared to young controls, but SFN treatment managed to maintain them at young values. In contrast, no significant changes in these cytokines were detected in the brains of adult male rats. SFN treatment did not alter their levels, which could explain the different effects in the two sexes. This is an example of how the same drug can have different effects in males and females.

Although our results are promising for the use of SFN as a protective agent, it has already emphasized the importance of “resisting the temptation” to extrapolate the results to humans when using short-term experimental model systems (Rattan 2024), so SFN should be evaluate in humans, both men and women, in order to take into account events that may modify the outcomes related to sex, such as menopause.

In summary, aging-related cognitive decline is caused trough different pathways in females and males. Females present higher inflammation levels causing an early cognitive decline, which is stablished during middle age, while males cognitive decline could be owed to an accumulation of senescent cells in the brain, leading to a gradual decline. Since glial cells, such as astrocytes and microglia, are intimately linked to neuroinflammation, it will be important in future studies to determine the effect of SFN on each cell type.

SFN prevented cognitive decline in both, females and males, during adulthood, but was not able to prevent it in old age. SFN lowered both, senescence and inflammation in cortex and hippocampus, preserving brain homeostasis. Further sex-specific studies, particularly in female animal models, are needed to understand how different drugs act in a sex-dimorphic manner.

## Supplementary Information

Below is the link to the electronic supplementary material.

Supplementary file1 (DOCX 31 KB)

## Data Availability

No datasets were generated or analysed during the current study.
