# Supplementary material for: Sex differences in middle-aged and old Wistar rats in response to long-term sulforaphane treatment for prevention of neuroinflammation, cognitive decline and brain senescence
Source: Biogerontology. 2025 May 17;26(3):110. doi: 10.1007/s10522-025-10231-0 (PMC12085354; doi:10.1007/s10522-025-10231-0)
Supplement: Supplementary file 1 — Supplementary file1 (DOCX 31 KB) [file 10522_2025_10231_MOESM1_ESM.docx]

| **Table 1A. Pro-inflammatory cytokines** | | | | | | | | | | |
| --- | --- | --- | --- | --- | --- | --- | --- | --- | --- | --- |
| **Cx** | FEMALE | | | | | MALE | | | | |
|  | Young | Adult | Adult  SFN | Old | Old  SFN | Young | Adult | Adult  SFN | Old | Old  SFN |
| G-CSF | 1509.38 ± 180.23 | 1491.57 ± 171.38 | 1397.43 ± 95.77 | 1313.69 ± 396.22 | 1536.55 ± 89.63 | 1609.46 ± 175.48 | 1528.77 ± 150.54 | 1408.85 ± 111.15 | 1541.57 ± 130.66 | 1430.19 ± 74.02 |
| GM-CSF | 267.43 ± 79.58 | 370.17 ± 82.50 | 269.86 ± 37.72  **b** | 315.28 ± 34.07 | 347.20 ± 57.24 | 310.62 ± 31.18 | 397.14 ± 74.60 | 313.66 ± 15.18  **b** | 326.35 ± 19.73 | 302.86 ± 68.02 |
| IFNγ | 209.83 ± 51.31 | 295.14 ± 36.24  **a** | 215.83 ± 28.04  **b** | 206.1 ± 19.17  **c** | 304.92 ± 76.41 | 206.93 ± 77.68 | 168.46 ± 64.00 | 220.75 ± 64.26 | 220.77 ± 52.02 | 209.08 ± 33.54  ***** |
| IL-1α | 2119.56 ± 351.95 | 3394.93 ± 706.60  **a** | 1819.08 ± 546.85  **b** | 2802.83 ± 302.36  **a, c** | 3377.48 ± 631.49  **a,c** | 1503.90 ± 609.68 | 2920.42 ± 761.93 **a** | 1554.47 ± 356.73 **b** | 2298.21 ± 477.25 | 1007.06 ± 278.31  **b, d*** |
| IL-1β | 838.84 ± 369.70 | 1973.16 ± 85.18  **a** | 1550.73 ± 99.19  **b** | 2582.69 ± 100.16 **a,b, c** | 2445.23 ± 280.71  **a, b,c** | 1315.19 ± 108.81 ***** | 937.68 ± 80.14  **a*** | 621.16 ± 238.36 **a,b *** | 1625.65 ± 135.89  **b,c *** | 1577.55 ± 245.34  **b, c *** |
| IL-17A | 400.28 ± 119.55 | 835.28 ± 199.96  **a** | 576.68 ± 135.91  **b** | 487.64 ± 48.43  **b** | 468.67 ± 108.15 | 309.79 ± 87.82 | 366.01 ± 130.97  ***** | 399.91 ± 130.59 | 640.66 ± 255.52 | 414.94 ± 53.76 |
| IL-2 | 292.49 ± 80.812 | 892.76 ± 142.44  **a** | 742.98 ± 72.41  **a** | 479.90 ± 208.71  **b** | 500.31 ± 115.72  **a, b** | 418.98 ± 189.91 | 255.95 ± 151.68  ***** | 350.62 ± 126.23  ***** | 450.94 ± 257.06 | 490.00 ± 301.09 |
| IL-5 | 174.25 ± 30.58 | 200.78 ± 70.63 | 226.32 ± 17.14 | 176.85 ± 36.508 | 235.69 ± 38.02 | 193.71 ± 33.624 | 169.55 ± 33.02 | 191.84 ± 38.43 | 197.94 ± 35.17 | 167.31 ± 4.768  ***** |
| IL-6 | 224.66 ± 81.55 | 362.55 ± 80.54  **a** | 254.29 ± 8.65  **b** | 250.63 ± 22.48 | 303.48 ± 87.51 | 233.8 ± 67.49 | 184.94 ± 66.25  ***** | 251.56 ± 49.80 | 204.10 ± 20.80  ***** | 157.86 ± 44.36  ***** |
| TNF-α | 430.96 ± 174.31 | 741.91 ± 93.95  **a** | 465.67 ± 73.63  **b** | 655.25 ± 35.24  **a, c** | 500.18 ± 92.58  **b** | 513.42 ± 193.95 | 543.8 ± 90.15 | 288.20 ± 24.24  **b*** | 656.88 ± 53.80 | 490.95 ± 206.42 |

| **Table 1B. Pro-inflammatory cytokines** | | | | | | | | | | |
| --- | --- | --- | --- | --- | --- | --- | --- | --- | --- | --- |
| **Hc** | FEMALE | | | | | MALE | | | | |
|  | Young | Adult | Adult | Old | Old | Young | Adult | Adult | Old | Old |
|  |  |  | SFN |  | SFN |  |  | SFN |  | SFN |
| G-CSF | 1445.22 ± 88.75 | 1771.41 ± 72.06  **a** | 1319.31 ± 59.68  **b** | 1796.02 ± 61.30 **a,c** | 1719.58 ± 22.55  **a,c** | 1465.62 ± 92.82 | 1823.67 ± 66.50  **a** | 1580.34  ±50.78  **b *** | 1785.01 ± 56.56  **a,c** | 1724.76 ± 37.04 **a,c** |
| GM-CSF | 217.43 ± 56.13 | 303.50 ± 69.13 | 303.2 ± 73.63 | 318.61 ± 36.46 | 307.20 ± 28.81 | 253.12 ± 51.69 | 297.14 ± 74.60 | 290.33 ± 45.09 | 303.02 ± 62.08 | 276.19 ± 54.66 |
| IFNγ | 155.83 ± 46.05 | 405.47 ± 20.67  **a** | 245.83 ± 73.87  **b** | 472.76 ± 45.35  **a,c** | 314.92 ± 37.16  **a,b, d** | 194.43 ± 61.48 | 321.79 ± 36.38  **a *** | 220.75 ± 64.26  **b** | 487.44 ± 25.41 **a,b,c** | 295.75 ± 25.87  **d** |
| IL-1α | 2089.80 ± 277.60 | 2658.52 ± 316.38 | 2331.10 ± 362.89 | 3241.20 ± 465.76 **a,c** | 3593.57 ± 527.54  **a,b,c** | 2999.16 ± 960.70 | 4980.62 ± 855.35  **a *** | 3250.72 ± 692.76 **b** | 3404.55 ± 554.77  **b** | 2809.95 ± 164.01  **b*** |
| IL-1β | 819.02 ± 134.19 | 3219.48 ± 680.76 **a** | 2019.14 ± 399.97 **a,b** | 3805.66 ± 567.60  **a,c** | 2445.23 ± 280.71  **a,d** | 1543.28 ± 348.21 ***** | 2832.48 ± 494.74 **a** | 1885.44 ± 213.24 **b** | 4197.12 ± 560.90  **a,b,c** | 3669.28 ± 478.63  **a,c** |
| IL-17A | 279.28 ± 39.0  5 | 668.61 ± 152.71  **a** | 513.35 ± 70.91  **a** | 587.64 ± 48.43  **a** | 468.67 ± 52.14  **a** | 276.46 ± 87.43 | 432.67 ± 90.00 | 466.57 ± 74.49  **a** | 540.66 ± 157.16  ***** | 481.60 ± 61.61  **a** |
| IL-2 | 305.38 ± 24.75 | 900.65 ± 78.72  **a** | 572.56 ± 45.82 **a,b** | 1036.93 ± 42.29 **a,c** | 992.78 ± 51.70  **a,c** | 210.34 ± 26.41  ***** | 485.93 ± 16.97  **a *** | 418.35 ± 35.51  **a *** | 1048.97 ± 53.69 **a,b,c** | 937.78 ± 45.23 **a,b,c *** |
| IL-5 | 332.54 ± 54.71 | 607.09 ± 42.20  **a** | 464.27 ± 55.84  **a,b** | 588.3 ± 37.25  **a,c** | 583.83 ± 60.34  **a,c** | 280.495 ± 26.65  ***** | 513.50 ± 58.91  **a *** | 431.84 ± 34.59  **a,b *** | 697.94 ± 35.17  **a,b,c** | 693.98 ± 79.13  **a,b,c** |
| IL-6 | 226.05 ± 35.06 | 450.68 ± 49.41  **a** | 272.94 ± 46.98  **b** | 412.18 ± 65.17  **a,c** | 461.75 ± 30.01  **a,c** | 210.45 ± 10.08  ***** | 324.25 ± 62.25  **a *** | 227.35 ± 31.77  **b *** | 414.95 ± 38.20  **a,c *** | 483.71 ± 99.29  **a,c** |
| TNF-α | 524.63 ± 127.19 | 793 ± 71.28  **a** | 539.61 ± 76.06  **b** | 888.58 ± 69.46  **a,c** | 900.18 ± 85.59  **a,c** | 538.42 ± 79.47 | 727.13 ± 104.45 | 321.85 ± 42.78 a,b ***** | 756.88 ± 52.26  **a,c *** | 657.62 ± 92.70  **c *** |

| **Table 2A. Anti-inflammatory cytokines** | | | | | | | | | | |
| --- | --- | --- | --- | --- | --- | --- | --- | --- | --- | --- |
| **Cx** | FEMALE | | | | | MALE | | | | |
|  | Young | Adult | Adult  SFN | Old | Old  SFN | Young | Adult | Adult  SFN | Old | Old  SFN |
| IL-10 | 2803.56 ± 884.71 | 3321.93 ± 475.29 | 4352.38 ± 489.86 **a,b** | 2453.01 ± 819.98  **c** | 3238.58 ± 711.71 | 2337.61 ± 424.87 | 2421.52 ± 119.67 | 2760.08 ± 246.66 ***** | 2646.33 ± 724.94 | 3118.56 ± 266.46 **a,b** |
| IL-12p70 | 1902.45 ± 908.70 | 2267.10 ± 866.83 | 2317.91 ± 219.56 | 2123.10 ± 200.25 | 2078.28 ± 226.50 | 2148.25 ± 643.20 | 1808.12 ± 530.16 | 1859.86 ± 280.25 | 1985.99 ± 335.83 | 1779.953 ± 285.79 |
| IL-13 | 326.32 ± 136.16 | 577.25 ± 85.46  **a** | 559.96 ± 64.53  **a** | 372.67 ± 131.39 | 397.91 ± 74.59 | 346.67 ± 114.95 | 261.97 ± 77.83  ***** | 295.14 ± 66.08  ***** | 346.65 ± 130.99 | 355.45 ± 162.83 |

| **Table 2B. Anti-inflammatory cytokines** | | | | | | | | | | |
| --- | --- | --- | --- | --- | --- | --- | --- | --- | --- | --- |
| **Hc** | FEMALE | | | | | MALE | | | | |
|  | Young | Adult | Adult  SFN | Old | Old  SFN | Young | Adult | Adult  SFN | Old | Old  SFN |
| IL-10 | 2963.56 ± 83.61 | 1287.65 ± 228.89 **a** | 3113.42 ± 229.38 **b** | 1554.24 ± 282.43  **a,c** | 1831.72 ± 335.25  **a,c** | 2571.95 ± 602.54 | 1511.21 ± 249.66 **a** | 2858.93 ± 417.77 **b** | 2129.67 ± 765.34 | 2119.55 ± 455.98 |
| IL-12p70 | 1622.45 ± 297.00 | 2600.43 ± 537.77 **a** | 2284.58 ± 163.38 **a** | 2789.77 ± 544.74 **a** | 2544.94 ± 381.89  **a** | 1698.25 ± 395.07 | 1841.45 ± 369.72 | 1993.2 ± 304.66 | 2319.33 ± 305.33 | 1946.62 ± 194.68 ***** |
| IL-13 | 525.47 ± 131.41 | 547.27 ± 51.87 | 539.95 ± 45.56 | 324.77 ± 93.84 **b,c** | 391.76 ± 74.27  **b,c** | 293.87 ± 58.68  ***** | 229.86 ± 36.67 ***** | 276.50 ± 53.96  ***** | 289.16 ± 52.11 | 395.56 ± 93.52 |

| **Table 3A. Chemokines** | | | | | | | | | | |
| --- | --- | --- | --- | --- | --- | --- | --- | --- | --- | --- |
| **Cx** | FEMALE | | | | | MALE | | | | |
|  | Young | Adult | Adult SFN | Old | Old SFN | Young | Adult | Adult SFN | Old | Old SFN |
|  |  |  |  |  |  |  |  |  |  |  |
| Eotaxin | 193.96 ± 56.14 | 318.60  ± 41.12  **a** | 242.46 ± 34.82  **b** | 311.88 ± 48.46 | 286.73 ± 26.15 | 198.99 ± 30.89 | 261.98 ± 26.87 | 233.92 ± 15.04  ***** | 299.55 ± 33.33  ***** | 280.52 ± 32.35 |
| Gro-α | 511.62 ± 71.72 | 831.22 ± 105.49  **a** | 609.52 ± 85.03 b **b** | 586.32 ± 79.73  **b** | 937.66 ± 298.49  **a** | 591.59 ± 116.09 | 522.46 ± 59.91  ***** | 573.44 ± 82.35  ***** | 584.83 ± 133.37 ***** | 528.72 ± 73.12  ***** |
| IP-10 | 1601.73 ± 455.10 | 3125.01 ± 511.31 **a** | 2663.19 ± 309.84  **a** | 1666.23 ± 653.95  **b,c** | 1831.99 ± 376.18  **b,c** | 1085.71 ± 397.03 | 955.80 ± 282.62 | 1276.86 ± 433.05 | 1363.46 ± 795.51 | 1421.34 ± 776.27 |
| MCP-3 | 167.62 ± 75.95 | 364.53 ± 78.19  **a** | 288.01 ± 44.76 | 224.31 ± 54.02 | 172.27 ± 98.50 | 132.34 ± 74.86 | 150.19 ± 71.78 | 82.63 ± 22.85  ***** | 335.53 ± 118.55  ***** | 264.2 ± 62.75  **c *** |
| MIP-1α | 417.13 ± 153.50 | 899.30 ± 179.78  **a** | 672.86 ± 65.90 **a,b** | 620.10 ± 41.73  **b** | 505.36 ± 76.78  **b** | 439.42 ± 171.61 | 367.58 ± 42.74 | 314.52 ± 93.07 | 346.85 ± 198.38 | 429.83 ± 80.53  ***** |
| RANTES | 8131.38 ± 1534.92 | 18207.07 ± 1554.96 **a** | 13729.88 ± 1166.17  **a,b** | 12388.72 ± 1361.05  **a,b** | 9076.97 ± 1472.87  **b,c,d** | 6846.19 ±  806.81  ***** | 5998.85 ± 1543.20  ***** | 6628.48 ± 1382.00 ***** | 13029.05 ±  500.93  ***** | 12536.02 ± 1942.18  **a,b,c *** |

| **Table 3B. Chemokines** | | | | | | | | | | |
| --- | --- | --- | --- | --- | --- | --- | --- | --- | --- | --- |
| **Hc** | FEMALE | | | | | MALE | | | | |
|  | Young | Adult | Adult SFN | Old | Old SFN | Young | Adult | Adult SFN | Old | Old SFN |
|  |  |  |  |  |  |  |  |  |  |  |
| Eotaxin | 276.42 ± 97.09 | 418.60 ± 41.12 | 345.8 ± 50.00 | 561.88 ± 60.99  **a,c** | 486.73 ± 26.15  **a,c** | 298.99 ± 61.76 | 495.31 ± 68.70  **a** | 400.59 ± 67.59  ***** | 632.88 ± 52.47  **a,c** | 513.86 ± 76.95 |
| Gro-α | 293.69 ± 58.88 | 784.55 ± 43.93  **a** | 676.18 ± 46.98  **a** | 752.98 ± 42.31  **a** | 697.85 ± 55.66  **a** | 301.59 ± 56.54 | 599.13 ± 60.66  **a** | 616.77 ± 63.31  **a** | 708.17 ± 43.25  **a** | 688.72 ± 28.57  **a *** |
| IP-10 | 367.73 ± 86.13 | 2925.01 ± 175.88 **a** | 3263.19 ± 422.14 **a** | 3266.23 ± 401.89 **a** | 2831.99 ± 641.06 **a** | 360.71 ± 87.46 | 2789.14 ± 603.61 **a** | 3276.86 ± 619.28 **a** | 3930.13 ± 722.23 **a** | 3154.67 ± 345.32 **a** |
| MCP-3 | 300.96 ± 40.12 | 731.19 ± 76.56 **a** | 601.34 ± 81.94 **a** | 770.97 ± 57.76 **a** | 729.94 ± 37.35 **a** | 302.34 ± 59.65 | 550.47 ± 48.87 **a** | 482.63 ± 22.85 **a** | 688.98 ± 47.88  **a,c** | 680.86 ± 51.49 **a,c** |
| MIP-1α | 455.03 ± 92.67 | 965.97 ± 102.14  **a** | 694.80 ± 51.97 **a,b** | 720.10 ± 58.28 a, **b** | 745.36 ± 84.43 **a,b** | 397.67 ± 84.85 | 680.91 ± 54.52  **a *** | 714.52 ± 30.78 **a** | 880.18 ± 50.13 **a,b,c *** | 729.83 ± 56.70  **a,d** |
| RANTES | 9006.38 ± 653.25 | 17540.40 ± 987.84 **a** | 14963.22 ± 486.75 **a,b** | 14122.05 ± 421.78  **a,b** | 15576.97 ± 568.62 **a,d** | 8346.19 ± 412.96 | 16932.19 ± 489.95 **a** | 15295.14 ± 592.58 **a,b *** | 19089.05 ± 586.84  **a,b,c *** | 18202.68 ± 531.68  **a,b,c *** |
